# A case series and literature review on 98 pediatric patients of germ cell tumor developing growing teratoma syndrome

**DOI:** 10.1002/cam4.6017

**Published:** 2023-05-04

**Authors:** Ming‐Yun Hsieh, Hsin‐Hung Chen, Chih‐Ying Lee, Giun‐Yi Hung, Tsung‐Yen Chang, Shih‐Hsiang Chen, Jin‐Yao Lai, Tang‐Her Jaing, Chao‐Neng Cheng, Jiann‐Shiuh Chen, Hsin‐Lin Tsai, Ting‐Yen Yu, Ming‐Hsin Hou, Cheng‐Yin Ho, Hsiu‐Ju Yen

**Affiliations:** ^1^ Department of Pediatrics Kaohsiung Veterans General Hospital Kaohsiung Taiwan; ^2^ School of Medicine, National Yang‐Ming Chiao‐Tung University Hsinchu Taiwan; ^3^ Department of Biological Sciences National Sun Yat‐Sen University Kaohsiung Taiwan; ^4^ Division of Pediatric Neurosurgery Neurological Institute, Taipei Veterans General Hospital Taipei Taiwan; ^5^ Department of Pediatrics Taipei Veterans General Hospital Taipei Taiwan; ^6^ Department of Life Science National Taiwan Normal University Taipei Taiwan; ^7^ Department of Pediatrics Chang Gung Memorial Hospital, School of Medicine, Chang Gung University Taoyuan Taiwan; ^8^ Department of Pediatric Surgery Chang Gung Memorial Hospital, School of Medicine, Chang Gung University Taoyuan Taiwan; ^9^ Department of Pediatrics National Cheng Kung University Hospital, College of Medicine, National Cheng Kung University Tainan Taiwan; ^10^ Division of Pediatric Surgery, Department of Surgery Taipei Veterans General Hospital Taipei Taiwan; ^11^ Department of Pediatrics Far Eastern Memorial Hospital New Taipei City Taiwan

**Keywords:** child, neoplasms, germ cell and embryonal, teratoma, event‐free survival

## Abstract

**Introduction:**

Malignant germ cell tumors (MGCTs) can develop either extracranially or intracranially. Growing teratoma syndrome (GTS) may develop in these patients following chemotherapy. Reports on the clinical characteristics and outcomes of GTS in children with MGCTs are limited.

**Methods:**

We retrospectively collected the data, including the clinical characteristics and outcomes of five patients in our series and 93 pediatric patients selected through a literature review of MGCTs. This study aimed to analyze survival outcomes and risk factors for subsequent events in pediatric patients with MGCTs developing GTS.

**Results:**

The sex ratio was 1.09 (male/female). In total, 52 patients (53.1%) had intracranial MGCTs. Compared with patients with extracranial GCTs, those with intracranial GCTs were younger, predominantly boys, had shorter intervals between MGCT and GTS, and had GTS mostly occurring over the initial site (all *p* < 0.001). Ninety‐five patients (96.9%) were alive. However, GTS recurrence (*n* = 14), GTS progression (*n* = 9), and MGCT recurrence (*n* = 19) caused a substantial decrease in event‐free survival (EFS). Multivariate analyses showed that the only significant risk factors for these events were incomplete GTS resection and different locations of GCT and GTS. Patients without any risk had a 5‐year EFS of 78.8% ± 7.8%, whereas those with either risk had 41.7% ± 10.2% (*p* < 0.001).

**Conclusion:**

For patients with high‐risk features, every effort should be made to closely monitor, completely remove, and pathologically prove any newly developed mass to guide relevant treatment. Further studies incorporating the risk factors into treatment strategies may be required to optimize adjuvant therapy.

## 1 INTRODUCTION

Germ cell tumors (GCTs) are a heterogeneous group of tumors that develop from the primordial germ cells. The histological types and sites of GCTs vary with age and sex. GCTs in pediatric patients can be found in the pineal region (6%), mediastinum (7%), retroperitoneum (4%), sacrococcygeal region (42%), ovary (24%), testis (9%), and other sites (8%).^1^ GCTs can be classified as benign or malignant based on histological variations in cell type and degree of subsequent differentiation. Mature teratomas exhibiting terminal somatic differentiation are considered benign, whereas immature teratomas (ITs) containing immature components, primarily neuroectodermal tissue, are considered malignant tumors. Malignant GCTs (MGCTs) of varying degrees of differentiation are classified into two categories: germinoma‐family tumors (e.g., testicular seminoma, ovarian dysgerminoma, and intracranial germinoma) and non‐germinomatous germ cell tumors (e.g., choriocarcinoma, yolk sac tumor, and embryonal carcinoma).^2^, ^3^ Mixed MGCTs (MMGCTs) consist of two or more histological subtypes of malignant GCTs, including immature teratomas.

MGCTs and immature teratomas are treated with surgical resection and multiagent chemotherapy, with or without radiotherapy, whereas mature teratomas are typically treated with surgical resection. Recurrence of any mass lesion during or after treatment is concerning due to unfavorable outcomes of recurrent MGCTs despite aggressive salvage therapy.^4^ In 1982, Logothetis et al^5^ discovered a rare condition called growing teratoma syndrome (GTS) in patients with stage III testicular GCT. GTS is characterized by an enlargement of metastatic masses during or after chemotherapy for GCT in the context of normal serum markers and only mature teratoma on histological analysis of the resected tumor specimen.

Several studies have documented GTS in patients with GCT, predominantly adults.^6^, ^7^ In contrast, only a few cases of GTS in pediatric patients have been reported. The number of studies on the GTS incidence rate, clinical features, and outcomes in children with GCTs is limited. In this study, we observed five pediatric patients with GTS following MGCTs admitted to three tertiary medical centers in Taiwan. After an extensive review of the literature, we identified a few instances of GTS in pediatric patients with MGCT and collected relevant information on their clinical characteristics, management, and treatment outcomes.

The objective of this study was to reveal survival outcomes of pediatric patients with MGCTs developing GTS and identify risk factors for subsequent events. The second objective was to clarify differences between patients with extra‐ or intra‐cranial MGCTs developing GTS, with a focus on timing of GTS development, treatment strategies, and outcomes.

## 2 METHODS

This was a retrospective study with secondary data analysis. First, we reviewed the medical records of 86 pediatric patients aged ≤18 years with MGCTs who were treated at three tertiary medical centers in Taiwan between 2006 and 2021. Among them, five patients were diagnosed with GTS. We screened data on patients' characteristics, histology subtypes, treatment of initial MGCTs, time of onset, and treatment of GTS, subsequent events, and current disease status.

**FIGURE 1:**
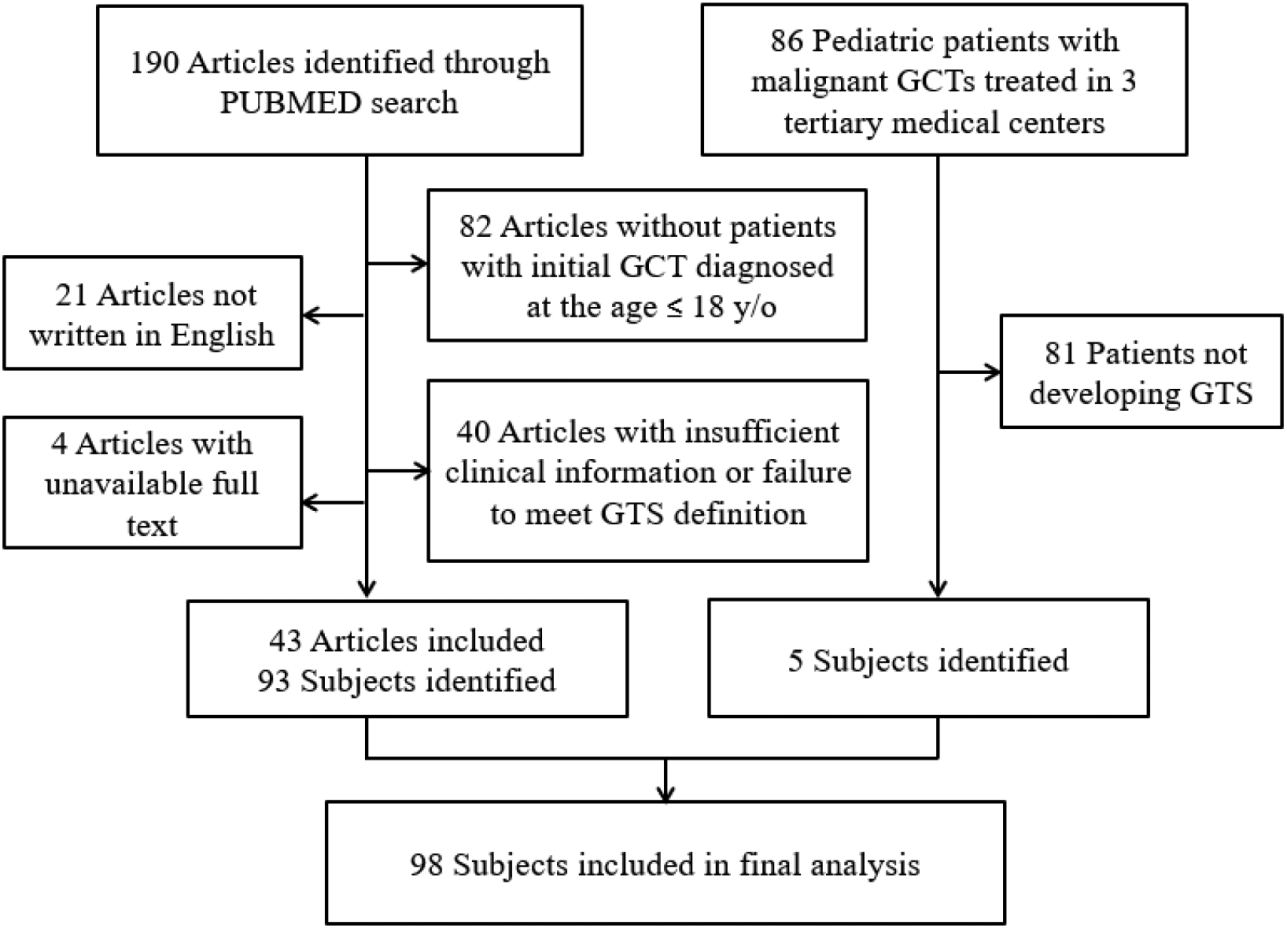
CONSORT flow diagram for article selection and case enrollment in this study.

Then, a literature review on GTS was conducted in PubMed using the keyword “growing teratoma syndrome” to search for articles published between January 1982 and December 2021. The exclusion criteria were as follows: (1) patients who received an initial GCT diagnosis at the age older than 18 years; (2) patients with insufficient clinical information or failure to meet the GTS definition defined by Logothetis et al^5^; (3) articles not written in English; and (4) unavailable full text. The article selection and enrollment details are listed in Figure 1. The study was approved by the Institutional Review Board (IRB) of the study hospital (TPVGH‐IRB 2023‐01‐014AC). A waiver of documentation for signed informed consent was also granted by the IRB.

After dividing the patients into intracranial and extracranial groups, we grouped them into pure IT and MGCT excluding pure IT. Therefore, four groups of patients were included in this study: (1) intracranial MGCT excluding pure IT (iMGCT), (2) intracranial and pure IT (iIT), (3) extracranial MGCT excluding pure IT (eMGCT), and (4) extracranial and pure IT (eIT).

We used the *t* test to compare median values and the chi‐square and Fisher's exact tests to compare frequency distributions. The Cox regression model was used to identify risk factors for events after GTS diagnosis. The backward elimination method (likelihood ratio) was used to select variables for inclusion in the multivariate analysis based on the results of univariate analyses with a threshold of *p* < 0.10.

The Kaplan–Meier method was used to estimate overall survival (OS) and event‐free survival (EFS) curves. Based on the benign nature of GTS reported in the literature, the occurrence of any event after the development of GTS was substantial. The events were defined as (1) GTS recurrence after complete resection of GTS; (2) GTS progression (i.e., enlargement of the mass) after incomplete resection of GTS; (3) GCT recurrence, with or without somatic transformation; (4) new mass lesions of unknown nature; and (5) death. The log‐rank test was used to compare survival curves. Statistical significance was set at *p* ≤ 0.05. All statistical analyses were performed using IBM SPSS Statistics (v16).

## 3 RESULTS

### 3.1 Patient characteristics and treatment details of five patients in our series

The details of GTS in the five pediatric patients with GCTs are listed in Table 1. Three boys and two girls with a mean age of 12.2 years at the initial diagnosis of GCT were included. One patient had intracranial MMGCT in the pineal region; among the four patients who had extracranial GCT, one had IT in the ovary, one had YST in the ovary, and two had MMGCT in the testis. All five patients received chemotherapy for GCT. However, one patient (case 4) with stage II right testicular malignant GCT received chemotherapy only after experiencing relapse over the retroperitoneum, which was identified as GTS during chemotherapy, and received postsurgical radiotherapy due to incomplete resection of GTS. As scheduled adjuvant treatment, the patient with intracranial GCT received radiotherapy.

**TABLE 1:** Patient characteristics and treatment details of five pediatric GCT patients with GTS in our series.

	Case 1	Case 2	Case 3	Case 4	Case 5
Age/gender at GCT diagnosis (year)	7/M	12/F	16/M	15/M	11/F
Germ cell tumor					
Histological type	Germinoma, IT	IT, grade III	MT, YST, EC	MT, YST, EC	YST
Location	Pineal region	Left ovary	Left testis	Right testis	Left ovary
Stage	M0	III	I	II	I
Tumor marker at diagnosis (AFP, ng/mL; HCG, mIU/mL)	550/35.6	1141/<2	119/78.2	74/10.3	174,050/<0.5
Management	B, VP shunt, C/T, R/T	Tumor resection, C/T	Tumor resection, C/T	Tumor resection	Tumor resection, C/T
Disease status prior to GTS	Disease‐free	Disease‐free	Disease‐free	Under chemotherapy after tumor recurrence (normal AFP, only image) over paraspinal and para‐aortic lymph node	Disease‐free
Interval between GCT and GTS (months)	48	4	4	10	87
Growing teratoma syndrome					
Location	Pineal region	Peritoneum and pelvis	Retrocaval and aortocaval space	Para‐aortic and paraspinal mass	Right ovary
Management	Complete resection	Complete resection	Complete resection	Incomplete resection, R/T	Complete resection
No. of GTS progression/recurrence	0	1	2	2	0
Interval (months)	–	8 months	4, 3 months	43, 56 months	–
Location of recurrent GTS	NA	Subphrenic and pelvic mass	Left paraaortic and retroperitoneum →right para‐aortic and retroperitoneum	Retroperitoneum→retroperitoneum	NA
Management upon later recurrences	NA	Complete resection^a^	Cryoablation→Cryoablation	Complete resection→Complete resection	NA
Follow‐up duration after GTS diagnosis (months)	8^b^	92	16	137	35
Current status	Loss to follow‐up	Disease‐free	Disease‐free	Disease‐free	Disease‐free

Abbreviations: AFP, alpha‐fetoprotein; B, biopsy; C/T, chemotherapy; EC, embryonal carcinoma; GCT, germ cell tumor; GTS, growing teratoma syndrome; HCG, human chorionic gonadotropin; IT, immature teratoma; MT, mature teratoma; NA, not applicable; R/T, radiation therapy; VP‐shunt, ventriculo‐peritoneal shunt; YST, yolk sac tumor.

^a^ Observation first at the diagnosis of recurrent GTS; resection 77 months later after recurrent GTS progressed.

^b^ A pancreatic tumor found 6 months later, then lost to follow‐up with unproved nature of the pancreatic tumor.

At the time of GTS diagnosis, four patients had completed their treatment for GCT and one was still undergoing scheduled chemotherapy. Four patients had complete resection of GTS, and one patient with residuals received adjuvant radiotherapy. During the follow‐up period, GTS recurred once in one patient (case 2), once in another patient after gross total resection of GTS (case 4), and twice in one patient (case 3). The only patient with intracranial GCT who had complete resection of GTS developed a pancreatic mass of unknown nature 6 months later and was lost to follow‐up. All patients were alive at the last follow‐up, and four of them were disease‐free.

### 3.2 Analyses of patient characteristics and treatment details of patients from literature search and our patients

A PubMed search with “growing teratoma syndrome” returned 190 articles, and 43 of them were eligible.^5^, ^8^, ^9^, ^10^, ^11^, ^12^, ^13^, ^14^, ^15^, ^16^, ^17^, ^18^, ^19^, ^20^, ^21^, ^22^, ^23^, ^24^, ^25^, ^26^, ^27^, ^28^, ^29^, ^30^, ^31^, ^32^, ^33^, ^34^, ^35^, ^36^, ^37^, ^38^, ^39^, ^40^, ^41^, ^42^, ^43^, ^44^, ^45^, ^46^, ^47^, ^48^, ^49^ In total, 93 identified patients from the literature review and our five patients were enrolled in the present study (Figure 1). Of these patients, 46 (47%) had extracranial GCTs (21 with eMGCTs and 25 with eITs), and 52 (53%) had intracranial GCTs (41 with iMGCTs and 11 with iITs). Table 2 shows the characteristics of the four subgroups. After excluding 36 patients with pure ITs, 57 (93.2%) of the 62 patients had mixed GCT components. Among them, three patients had pure germinoma and two patients had YST. All 46 patients with extracranial GCT received operations for GCT removal; 35 (76.1%) of them received adjuvant chemotherapy and/or radiotherapy at the initial diagnosis, 11 (23.9%) of them had adjuvant therapy after GCT recurrence, and 19 (36.5%) of 52 intracranial GCT patients did not receive GCT resection. However, all the patients received adjuvant chemotherapy and/or radiotherapy.

**TABLE 2:** Patient characteristics and treatment details of 98 pediatric GCT patients with GTS in the literatures and our series.

Patients' characteristics	Intracranial			Extracranial			Extracranial vs. intracranial
	iIT (*n* = 11)	iMGCT (*n* = 41)	iIT vs. iMGCT	eIT (*n* = 25)	eMGCT (*n* = 21)	eIT vs. eMGCT	
Median age (range, year)	9.8 (0.1–16)	10.5 (0.2–17)	0.829	12.9 (4–18)	12.7 (2–18)	0.917	0.001
Gender (M/F)	9/2	35/6	1.000	0/25	7/14	0.002	0.000
Location							
Pineal	9	31	1.000	–	–	0.002	NA
Sella/suprasella	2	7		–	–		
Ovary	–	–		25	14		
Testis	–	–		–	6		
Mediastinum	–	–		–	1		
Others	–	3		–	–		
Stage							
I/II	NA	NA	NA	12	11	1.000	NA
III/IV	NA	NA		13	10		
Management for GCT							
Operation			0.000			1.000	0.000
Complete resection	–	1		13^a^	11		
Incomplete resection(including biopsy and VP shunt placement)	11	21		12	10		
No	–	19		–	–		
Adjuvant therapy							
Chemotherapy and/or radiotherapy	11	41	NA	25 (8 of 25 after IT recurrence)	21 (3 of 21 after MGCT recurrence)	NA	NA
Interval between GCT and GTS (months)							
Median	3.3	7.6	0.463	12.8	15.8	0.465	0.000
Range	(1.5–5)	(1–48)		(2.5–108)	(2–87.5)		
GTS over primary GCT site							
Yes	11	39	1.000	16	13	1.000	0.000
No	–	2		9	8		
Management for GTS							
Operation							
Complete resection	9	33	1.000	17	16	0.744	0.344
Incomplete	2	8		8	5		
Adjuvant therapy after GTS							
Chemotherapy and/or radiotherapy	5^b^	24^b^	0.495	1	2	NA	0.000
No	6	16		21	17		
Other therapy	–						
Tretinoin, tramadol and sorafenib	–	–		1	–		
Interferon 2β and gamma knife	–	–		1	–		
Interferon 2β and tamoxifen	–	–		1	–		
Palbociclib	–	1		–	–		
Cryotherapy	–	–		–	2^c^		
Recurrence of GTS							
Total 2 episodes	1	–	0.518	7	2	0.502	0.003
Total 3 episodes	–	1		1	2		
GTS progression after first incomplete resection of GTS	–	2	1.000	3	4	0.686	0.079
Recurrence of MGCT, including somatic transformation/unknown mass							
Before GTS	–	–	1.000	8	4	0.744	0.044
After GTS	1	5		–	1		
Current status							
No evidence of disease	9	31	0.761	19	19	0.260	0.331
Stable disease	1	7		6	2		
Death	1^d^	2^e^		–	–		
Unknown	–	1^f^		–	–		
Follow‐up duration following GTS diagnosis (months)							
Median	24.5	51.7	0.090	46.6	69.5	0.285	0.738
Range	(4–62)	(4–141)		(3–177)	(1–190)		

Abbreviations: B, biopsy; C/T, chemotherapy; EC, embryonal carcinoma; eIT, extracranial pure immature teratoma; eMGCT, extracranial malignant germ cell tumor; GCT, germ cell tumor; GTS, growing teratoma syndrome; IT, immature teratoma; iIT, intracranial pure immature teratoma; iMGCT, intracranial malignant germ cell tumor; MT, mature teratoma; NA, not applicable; R/T, radiation therapy; VP‐shunt, ventriculo‐peritoneal shunt; YST, yolk sac tumor.

^a^ One with unknown post‐operation status.

^b^ For GTS development during adjuvant treatment for primary GCT.

^c^ Both receiving cryotherapy after GTS progression/recurrence.

^d^ Died of drug toxicity of chemotherapy and poor general condition after GCT recurrence.

^e^ Carcinomatous change of GCT over the primary GCT site and deteriorated later in 1; died of GTS progression in the other.

^f^ One developing a pancreatic tumor with unknown diagnosis.

Compared with those of extracranial groups, the patients of intracranial groups were younger (10.4 vs. 12.8 years old, *p* = 0.001), predominantly male (84.6% vs. 15.2%, *p* < 0.001), had shorter median intervals between GCT and GTS (6.7 vs. 14.2 months, *p* < 0.001), and were more likely to have GTS over the primary GCT site (96.2% vs. 63.0%, *p* < 0.001).

### 3.3 GTS management and further recurrence of GTS and GCT

Surgical resection was attempted in all patients with incomplete resection, which included 23 (23.5%) patients, specifically 10 (19.2%) of 52 intracranial patients and 13 (28.3%) of 46 extracranial patients (*p* = 0.344). After the diagnosis of GTS, 29 (55.8%) patients in the intracranial group resumed adjuvant therapy for primary GCT, whereas only three (6.5%) patients in the extracranial group received additional chemotherapy and/or radiotherapy. Other three (6.5%) patients in the extracranial group received other medical therapies because of incomplete GTS resection.

GTS recurrence and progression were observed in 14 and 9 patients, respectively. Of the 14 patients with GTS recurrence, three in the extracranial group and one in the intracranial group had three episodes of GTS. Two patients received cryotherapy after GTS recurrence or progression of incompletely resected GTS and reached stable disease status afterward.

In addition to subsequent GTS episodes, 19 (19.4%) patients had GCT recurrence, of whom one had somatic malignant transformation and the other had a pancreatic mass of unknown nature; both were in the intracranial group. Among these 19 patients, 13 (68.4%) patients were in the extracranial group and 6 were in the intracranial group, respectively. Before the diagnosis of GTS, GCT recurrences occurred in 12 (92.3%) of the 13 extracranial patients; however, after the diagnosis of GTS, GCT recurrences occurred in all six intracranial patients.

### 3.4 Outcome and risk factors for events after diagnosis of GTS

**FIGURE 2:**
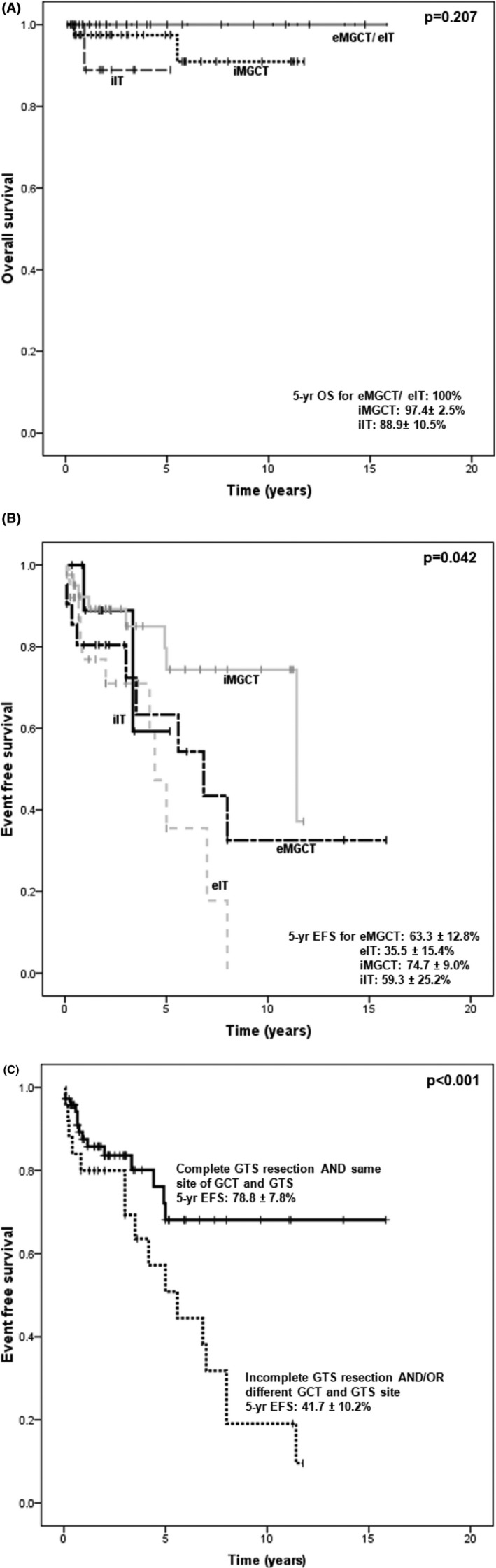
Overall survival curve (A), and event‐free survival curve (B) among intracranial and extracranial subgroups in pediatric GCT patients developing GTS. (C) Event‐free survival rates between patients with complete resection AND same site of GTS and GCT, and patients with incomplete resection AND/OR different site of GTS and GCT. eIT, extracranial pure immature teratoma; eMGCT, extracranial malignant germ cell tumor excluding pure immature teratoma; iIT, intracranial pure immature teratoma; iMGCT, intracranial malignant germ cell excluding pure immature teratoma.

At a median follow‐up time of 51.2 months after GTS diagnosis, 95 (96.9%) patients were alive, with 78 (79.6%) in complete remission, 16 (16.3%) in stable residual disease, and 1 lost to follow‐up. The 5‐year OS rates for iIT, iMGCT, eIT, and eMGCT were 88.9% ± 10.5%, 97.4% ± 2.5%, 100%, and 100%, respectively (*p* = 0.204, Figure 2A). Of the three expired patients, one in the iIT group died from treatment toxicity and the other two in the iMGCT group died from GCT recurrence and GTS progression, respectively. The 5‐year EFS rates (Figure 2B) for iIT, iMGCT, eIT, and eMGCT were 59.3% ± 25.2%, 74.7% ± 9.0%, 35.5% ± 15.4%, and 63.3 ± 12.8%, respectively (*p* = 0.042).

The significant risk factors for events after GTS diagnosis were evaluated through univariate analyses (*p* < 0.05) including extracranial GCT, histology of IT at the GCT diagnosis, incomplete resection of the initial GCT, incomplete GTS resection, and site of the initial GCT and GTS. However, only incomplete resection of GTS (hazard ratio [HR] = 3.069, *p* = 0.003) and different sites of GTS and GCT (HR = 4.707, *p* = 0.001) were statistically significant according to the multivariate analysis. The results of the Cox regression analyses are shown in Table 3. The estimated 5‐year EFS for patients with complete GTS resection and the same location of GTS and initial GCT was 78.8% ± 7.8%, while patients with incomplete GTS resection or/and at different sites of GTS and GCT had a 5‐year EFS of 41.7% ± 10.2% (*p* < 0.001) (Figure 2C).

**TABLE 3:** Analysis of risk factors for events^a^ in pediatric GCT patients with GTS.

Variables	Univariate HR (95% CI)	*p* value	Multivariate HR (95% CI)	*p* value
Age				
>10 vs. ≦10 years^b^	1.131 (0.753–1.697)	0.553	–	–
>5 vs. ≦5 years^b^	0.703 (0.573–1.200)	0.203	–	–
Gender male vs. female^b^	0.829 (0.328–1.440)	0.321	–	–
GCT stage^c^				
High (III/IV) vs. low (I/II)^b^	0.509 (0.183–1.415)	0.196	–	–
Location				
Extracranial vs. intracranial^b^	2.461 (1.151–5.260)	0.020	1.327 (0.478–3.686)	0.587
Gonad vs. non‐gonad^b^	1.461 (1.007–2.118)	0.046	–	–
Histology				
IT vs. MGCT^b^	2.202 (1.032–4.700)	0.041	1.638 (0.759–3.537)	0.209
Interval between GCT and GTS				
>3 vs. ≤3 m^b^	1.337 (0.873–2.046)	0.182	–	–
Resection of GCT				
Incomplete vs. complete^b^	0.445 (0.207–0.959)	0.039	0.187 (0.021–1.652)	0.131
Resection of GTS				
Incomplete vs. complete^b^	2.696 (1.302–5.582)	0.008	3.069 (1.463–6.439)	0.003
Adjuvant therapy after GTS				
No vs. yes^b^	0.863 (0.598–1.248)	0.434	–	–
Same site of GTS and GCT				
No vs. yes^b^	3.509 (1.512–8.146)	0.003	4.707 (1.954–11.335)	0.001

^a^ Events include growing teratoma syndrome (GTS) recurrence/progression, germ cell tumor (GCT) recurrence with somatic transformation or not, mass of unknown nature, or death.

^b^ Reference group.

^c^ Only for extracranial MGCT.

## 4 DISCUSSION

Although the incidence of GTS in intracranial GCT patients is reported to be 5% (39 out of 777 patients) and 6.5% (6 out of 52 patients), respectively,^22^, ^36^ data on the incidence of GTS in extracranial GCT patients are not available. However, the frequency of GTS in testicular non‐seminomatous GCT (NSGCT) and ovarian pure or mixed IT was reported to be 1.9%–7.6%^6^, ^11^, ^50^ and 12%–20%,^38^, ^51^ respectively. In our small series, the incidence of GTS among patients with GCT, excluding mature teratoma, diagnosed <18 years of age, was 5/86 (5.8%).

According to our literature review, the incidence rate of GTS patients with ovarian IT supports the fact that GTS is more common in patients with ovarian IT, with 35.6% of female patients with MGCTs having immature teratoma.^52^ Among 98 GTS patients in this review, 25 (25.5%) had MGCTs originating from ovarian IT, 11 (11.2%) from the intracranial IT, and 62 (63.3%) from either the intracranial or extracranial MGCT excluding pure IT. MT or IT components in pathology‐proven biopsy samples or resected tumors were identified in 13 (76.5%) of 17 patients with iMGCT and 19 (95%) of 20 patients with eMGCT, respectively. In a study of 39 patients with intracranial GTS,^22^ MT or IT was identified in 15 (93.8%) of the 16 surgical samples. In addition, another study found MT in 24 out of 28 (86%) primary GCTs among 30 male patients with NSGCT who developed GTS.^6^ Our findings are consistent with previous studies, suggesting that patients who have either IT or MGCT with an MT/IT component at diagnosis are more likely to develop subsequent GTS. These findings corroborate the hypothesis that GTS is induced by chemotherapeutic retroconversion of malignant components and contributes to mature teratoma growth.^41^ However, GTS development in a few patients with MGCTs who did not have MT/IT may be related to sampling bias due to tumor heterogeneity or chemotherapy‐altered cell transformation from a totipotent malignant germ cell to a benign mature teratoma.^53^

In our study, patients with intracranial GCT had a significantly shorter interval between the diagnosis of GTS and the initial GCT (6.7 vs. 14.1 months, *p* < 0.001). Michaiel et al^22^ reported early occurrence of GTS in the intracranial GCT with a median time of 2 months. By contrast, the median interval between GTS and initial GCT was longer than 20 months in 21 of 30 patients with initial testicular/mediastinum NSGCT following GTS occurrence after the completion of chemotherapy.^6^ The corresponding interval was 18.5 months in 35 GTS patients with ovarian IT.^51^ The difference in the interval is consistent with the findings of the previous studies and ours, possibly because of the high percentage of nonresection of GCT at initial diagnosis in patients of the intracranial group.

Nineteen patients (19.4%) among 98 GCT patients had GCT recurrence. All six patients with intracranial GCT had recurrences after the diagnosis of GTS, and 12 of 13 patients with extracranial GCT had recurrences before the diagnosis of GTS. Nine of 12 patients with extracranial MGCT had GCT recurrence before GTS diagnosis (ovarian IT:2 grade I, 1 grade II, and 3 grade III; eMGCT:2 stage I and 1 stage II) and did not receive adjuvant chemotherapy until GCT recurrence, which not only reflects the importance of systemic therapy for malignant GCT but also the inconsistent opinion on adjuvant chemotherapy for ovarian IT.^54^

Among the 52 patients in the intracranial group, two (3.8%) and six (11.5%) had GTS or GCT recurrence, respectively. Among the 46 patients in the extracranial group, 12 (26.1%) and 13 (28.3%) had GTS or GCT recurrence, respectively. Despite the fact that extracranial groups had a higher chance of having GTS or GCT recurrences (35.5% and 63.3% of 5‐year EFS in eIT and eMGCT, respectively), their 5‐year OS was comparable to that of intracranial patients, which indicates a relatively benign nature of GTS and good salvage rate for extracranial GCT despite recurrence. The risk for subsequent GCT or GTS recurrence was substantial in both groups, which highlights the necessity for surgical removal to clarify the nature of the growing mass, and vigilant awareness of GTS in patients with GCTs may guide pediatric hematologists and oncologists to intervene promptly in case of GCT recurrence and avoid unnecessary chemotherapy for GTS.

In our study, we identified the major risk factors for events after GTS diagnosis: incomplete resection of GTS and different locations of GTS and GCT. Andre et al^6^ described that one of 24 (4%) male patients with NSGCT undergoing complete resection of GTS experienced recurrence, whereas five of six (83%) male patients with NSGCT with incomplete resection of GTS experienced recurrence. Our findings are consistent with these findings, and complete resection of GTS if feasible is highly recommended. According to our literature review, sites of GTS differing from the initial GCT site have not yet been recognized as a risk factor for events after GTS diagnosis. Because a lower 5‐year EFS of 41.7% was observed in patients with incomplete resection of GTS and/or different sites of GTS and GCT, additional studies are warranted to design an optimal adjuvant therapy for these patients. Currently, the mainstay treatment for GTS is complete resection.^55^

While complete removal is unfeasible, therapies other than chemotherapy and/or radiotherapy have been used as adjuvant therapy. Postovsky et al^47^ reported a stable disease status induced by interferon α‐2B after the diagnosis of GTS following an intra‐abdominal large IT, grade 0–1 with disseminated tumorous nodules, on a 14‐year‐old girl. Hsieh et al^35^ reported a reduced‐size residual mature teratoma after resection in a 4‐year‐old girl with IT, grade 3 in the left ovary initially, slow regression of gliomatosis peritonei and ascites after the use of interferon α‐2B for 9 months, followed by gamma knife surgery 3 years after interferon discontinuation. A regimen of temozolomide 40 mg/m PO daily (days 1–28), tretinoin 25 mg/m/day PO BID (days 1–14), and sorafenib 150 mg/m^2^ PO BID (days 1–28), with the addition of thalidomide from the second cycle was given to a 12‐year‐old girl with a grade 3 FIGO stage IIIC immature teratoma in the right ovary and incomplete resection of GTS in the abdomen, pelvis, and chest. She had stable disease for 4 months on the preceding regimen.^44^ Stabilization of extracranial GTS in three men, aged 22–37 years, was observed with the use of palbociclib, a selective inhibitor of the cyclin‐dependent kinases CDK4 and CDK6.^56^ Schultz et al^12^ adopted palbociclib to treat intracranial GTS progression of a 5‐year‐old child with pineal GCT after incomplete resection of GTS. After 22 cycles of palbociclib, 75 mg, on days 1–21 of each 28‐day cycle, the condition of the child's GTS was stable. In addition, one patient with eMGCT received cryotherapy with successful local control for multiple GTS at different sites. Cryotherapy has also been reported to stabilize GTS over the liver surface in another pediatric patient.^8^ Therefore, local control by cryotherapy may provide another treatment option when complete surgical resection is difficult.

Owing to the relative rarity of GTS, no published report has compared the characteristics of pediatric and adult GTS. According to Hiester et al^55^ and Wang et al,^51^ the mean ages of patients with testicular GCT and ovarian GCT at the time of GTS diagnosis were 21–33 and 20–29 years, respectively. In contrast to patients with testicular GCT, who ranged in age from 16 to 38 years, patients with ovarian GCT ranged in age from 8 to 48 years old. The findings are comparable to those of our review; the majority of extracranial GTS resulted from ovarian GCT (pure IT or MGCT excluding pure IT), and substantially fewer occurred in extracranial GTS in male adolescents. Furthermore, the distribution of histology of primary GCT in these reviews was similar to that in our review. Due to the rarity of intracranial GTS in adults,^57^ a comparison of intracranial GTS between pediatric and adult groups is difficult.

Considering the epidemiology of intracranial GCT in children, the incidence peaks in infants (0–2 years) and adolescents (13–19 years).^58^ The extracranial counterpart peaks in young children (0–4 years) and increases from puberty onset through young adulthood (11–35 years).^59^ Both factors help explain the younger age of the intracranial group in our study (10.4 vs. 12.8 years old, *p* = 0.001). Although extracranial GCT patients of younger age (<12 years) are reported to have better disease‐free survival than the older,^59^ the prognosis of intracranial GCT patients is associated with different histological types and posttreatment response.^58^ In our study, age was not a risk factor for events in either intracranial or extracranial GCT patients experiencing GTS.

The limitations of this study are as follows: (1) it was a retrospective study involving patient data collection based on the literature review through a PUBMED search. Because a modest number of patients were excluded from analyses for insufficient clinical information, the results may not be generalizable to the whole population due to possible selection bias. (2) We enrolled patients being treated within 40 years, during which the standards of treatment, including use of chemotherapeutic agents, radiation policy, and surgical approach, varied, which may further impact on survivals due to lack of homogeneity. However, the differences in age, sex, and timing for the sequential development of GTS and GCT between the intracranial and extracranial groups are evident.

## 5 CONCLUSION

Either GTS or GCT can develop during or after chemotherapy in pediatric patients with intracranial or extracranial GCTs. Patients with intracranial GTS tend to be younger, and the interval between the initial GCT and GTS is shorter than in patients with extracranial GTS. Despite the low mortality rate, events from GTS, GCT recurrence, or somatic transformation following GTS diagnosis are common and can occur years after GTS diagnosis. In addition to complete surgical resection, GTS development at sites other than the initial GCT site poses a statistically significant risk for events. Further studies incorporating these risk factors into the treatment plan may help to guide optimal adjuvant therapy upon incomplete GTS resection.

## AUTHOR CONTRIBUTIONS

**Ming‐Yun Hsieh:** Writing – original draft (lead). **Hsin‐Hung Chen:** Conceptualization (equal); writing – original draft (supporting). **Chih‐Ying Lee:** Data curation (equal); formal analysis (equal). **Giun‐Yi Hung:** Data curation (equal); formal analysis (equal). **Tsung‐Yen Chang:** Data curation (equal); formal analysis (equal). **Shih‐Hsiang Chen:** Conceptualization (equal); writing – original draft (supporting). **Jin‐Yao Lai:** Investigation (equal); resources (supporting). **Tang‐Her Jaing:** Data curation (equal); formal analysis (equal). **Chao‐Neng Cheng:** Conceptualization (equal); writing – original draft (supporting). **Jiann‐Shiuh Chen:** Data curation (equal); formal analysis (equal). **Hsin‐Lin Tsai:** Investigation (equal); resources (supporting). **Ting‐Yen Yu:** Investigation (equal); resources (supporting). **Ming‐Hsin Hou:** Investigation (equal); resources (supporting). **Cheng‐Yin ho:** Investigation (equal); resources (supporting). **Hsiu‐Ju Yen:** Conceptualization (lead); resources (lead); supervision (lead); validation (lead); writing – review and editing (equal).

## FUNDING INFORMATION

The authors declare that there is financial support to Dr. Hsiu‐Ju Yen by grants from the Ministry of Health and Welfare, Taiwan (MOHW112‐TDU‐B‐222‐124017), and Taipei Veterans General Hospital, Taiwan (V112D68‐003‐MY3‐1).

## CONFLICT OF INTEREST STATEMENT

The authors declare that there is no conflict of interest.

## ETHICS STATEMENT

The study was approved by the Institutional Review Board (IRB) of the study hospital (TPVGH‐IRB 2023‐01‐014AC). A waiver of documentation for signed informed consent was also granted by the IRB.

## Data Availability

The data that support the findings of this study are available from the corresponding author upon reasonable request.
